# ROS-Scavenging Enzymes as an Antioxidant Response to High Concentration of Anthracene in the Liverwort *Marchantia polymorpha* L

**DOI:** 10.3390/plants10071478

**Published:** 2021-07-19

**Authors:** Nahuel Spinedi, Romina Storb, Elisabet Aranda, Facundo Romani, Maya Svriz, Santiago A. Varela, Javier E. Moreno, Sebastian Fracchia, Juan Cabrera, Ramón Alberto Batista-García, Inés Ponce de León, J. Martín Scervino

**Affiliations:** 1 Instituto de Investigaciones en Biodiversidad y Medioambiente (INIBIOMA), CONICET-UNCo, SC Bariloche, Río Negro 8400, Argentina; naspinedi@comahue-conicet.gob.ar (N.S.); storbromina@gmail.com (R.S.); mail.jcabrera@gmail.com (J.C.); 2 Institute of Water Research, University of Granada, Ramón y Cajal, 4, Bldg. Fray Luís, 18071 Granada, Spain; earanda@ugr.es; 3 Instituto de Agrobiotecnología del Litoral, UNL-Conicet, Facultad de Bioquímica y Ciencias Biológicas, Santa Fe 3000, Argentina; romani.facundo@gmail.com or; 4 Department of Plant Sciences, University of Cambridge, Downing Street, Cambridge CB2 3EA, UK; 5 Instituto de Investigaciones en Recursos Naturales, Agroecología y Desarrollo Rural (IRNAD), Universidad Nacional de Río Negro, CONICET, SC Bariloche, Río Negro 8400, Argentina; mayasvriz@gmail.com; 6 Grupo de Ecología Forestal, Instituto Nacional de Tecnología Agropecuaria (INTA) EEA Bariloche, CC 277, Bariloche 8400, Argentina; santiago.varela34@gmail.com; 7 Centro Regional de Investigaciones Científicas y Transferencia Tecnológica de La Rioja (CRILAR), UNLAR, SEGEMAR, UNCa, CONICET, Entre Ríos y Mendoza, 530 Anillaco, La Rioja 5300, Argentina; sebafrac@yahoo.com.ar; 8 Centro de Investigación en Dinámica Celular, Instituto de Investigación en Ciencias Básicas y Aplicadas, Universidad Autónoma del Estado de Morelos, Ave Universidad 1001, Col. Chamilpa, CP 62209 Cuernavaca, Morelos, Mexico; rabg@uaem.mx; 9 Departamento de Biología Molecular, Instituto de Investigaciones Biológicas Clemente Estable, Avenida Italia 3318, CP 11600 Montevideo, Uruguay; iponcetadeo@gmail.com

**Keywords:** antioxidant enzymes, peroxidases, liverwort, polycyclic aromatic hydrocarbons, anthracene, photosynthesis, RNA-sequencing

## Abstract

*Marchantia polymorpha* L. responds to environmental changes using a myriad set of physiological responses, some unique to the lineage related to the lack of a vascular- and root-system. This study investigates the physiological response of *M. polymorpha* to high doses of anthracene analysing the antioxidant enzymes and their relationship with the photosynthetic processes, as well as their transcriptomic response. We found an anthracene dose-dependent response reducing plant biomass and associated to an alteration of the ultrastructure of a 23.6% of chloroplasts. Despite a reduction in total thallus-chlorophyll of 31.6% of Chl *a* and 38.4% of Chl *b*, this was not accompanied by a significant change in the net photosynthesis rate and maximum quantum efficiency (*Fv/Fm*). However, we found an increase in the activity of main ROS-detoxifying enzymes of 34.09% of peroxidase and 692% of ascorbate peroxidase, supported at transcriptional level with the upregulation of ROS-related detoxifying responses. Finally, we found that *M. polymorpha* tolerated anthracene-stress under the lowest concentration used and can suffer physiological alterations under higher concentrations tested related to the accumulation of anthracene within plant tissues. Our results show that *M. polymorpha* under PAH stress condition activated two complementary physiological responses including the activation of antioxidant mechanisms and the accumulation of the pollutant within plant tissues to mitigate the damage to the photosynthetic apparatus.

## 1. Introduction

Anthropogenic pollution increasingly provokes deleterious impacts in all ecosystems, and particularly in soils, agriculture, mining, or domestic and industrial activities release a myriad of organic and inorganic pollutants, such as heavy metals, pesticides, pharmaceutical compounds and hydrocarbons, among others [1].

These substances are xenobiotic compounds that are not easily degraded by natural mechanisms, causing their accumulation in soils and the consequent radical increase of abiotic stresses in natural ecosystems [2]. Numerous investigations have approached this problematic issue from different points of view, such as using phytoremediation to reduce the phytoavailability of pollutants and to contribute to their reduced chance to get into the food chain, microbial remediation or the use of soil amendments to reduce their impact [3,4]. Polycyclic aromatic hydrocarbons (PAHs) are persistent organic pollutants that enter into the soil ecosystem primarily via atmospheric deposition. The concentration of these substances in the environment varies according to several parameters, such as pH, soil particle size, and presence of organic matter or lipophilic material, which tends to absorb PAHs into the soil. Anthracene, the structurally simplest PAH, can be found in nature (up to 504 µM on coal tar) [5,6,7].

PAH toxicity has been thoroughly studied in vascular plants, including some species of amaranth, poplar, pines and soybeans; there are a number of strategies used by plants to absorb, accumulate and detoxify chemical pollutants, showing different tolerance thresholds to PAHs [8,9,10]. Among the plant strategies used to cope with PAHs are: (1) accumulation and isolation of the pollutant within specialised organelles to limit toxicity or (2) activation of a detoxification response [11,12]. However, regardless of the strategy, the toxicity on vascular plants of different PAH concentrations causes different physiological responses and effects on organs depending on the duration, intensity and type of exposure. For example, in vitro experiments using Arabidopsis thaliana show the existence of a rapid oxidative stress response when exposed to 250 µM and 1250 µM of phenanthrene [13]. Among the reported effects to this exposure are root necrosis, leaf chlorosis, altered trichome formation, delayed flowering, inhibition of photosynthesis, oxidative stress and stunted growth [11,13,14,15,16,17].

The PAHs are widespread and accumulate at high levels in coastal areas, wetlands, and riverbanks. These habitats can be occupied by liverwort plants, such as *Marchantia polymorpha* L. whose ability to accumulate and detoxify these compounds is largely unknown. Unlike vascular plants, bryophytes are short creeping plants with intimate contact with the soil. Therefore, in the case of the thalloid liverwort *M. polymorpha*, the cells absorb water, nutrients and pollutants directly from the soil, which means a strong selection for tolerance and plasticity to different stressors [18].

Hence, it is important to investigate the physiological responses of liverwort grown in such habitats, to understand better the biochemical and molecular mechanisms by which liverwort counteracts the presence of high levels of PAH in the environment. It has been proved that *M. polymorpha* plants complete their life-cycle growth in concentrations of anthracene ranging from 50 to 280 µM [19]. Anthracene toxicity induced apparent phenotypes such as stunted growth with a marked reduction in dry weight and chlorophyll content index. Higher concentrations than 280 µM proved to be lethal for this plant. However, this was the first approach to understand the potential capacity of liverworts to accumulate and detoxify anthracene; at the biochemical and molecular level there are still several questions that remain unresolved.

A natural cytotoxic by-product of photosynthetic activity is the production of reactive oxygen species (ROS), including H_2_O_2_, O_2_^−^ and HO· that paradoxically serve as signalling molecules [20,21]. Given the alteration of the CO_2_ fixation or the inhibition of the Calvin cycle, the ROS in the chloroplast tend to increase, and upon certain conditions become toxic to the cell since they can oxidize cellular components that the antioxidant machinery cannot repair. Because of this, plant cells respond by means of highly controlled mechanisms to neutralize these compounds, including enzymatic and buffering mechanisms [22,23]. The principal abiotic stress triggered by PAHs is an oxidative stress induced by high levels of ROS resulting in programmed defence responses [24,25]. The accumulation of PAHs in the membrane activates the generation of antioxidant enzymes, principally SOD, to mitigate stress [24,25]. Plants have evolved ROS-detoxifying enzymatic machineries to minimize ROS-deleterious impacts. Mainly, there are three enzymes that work in tandem in the process of detoxification: superoxide dismutase (SOD), catalase (CAT) and peroxidase (POD). If the production of antioxidants is not enough, the excess of ROS usually reduces photosynthesis and respiration [13]. On the other hand, a plant with a robust antioxidant enzymatic system can likely sustain the rates of these central metabolic processes.

In this study, *M. polymorpha* L. plants exposed to anthracene were used to understand the physiological and molecular responses of the plant. In addition, the activity of the enzymes (SOD, CAT, POD and APX) related to the stress induced on the photosynthesis process were studied. Finally, we performed a transcriptomic study to analyse the transcriptomic landscape under an anthracene exposure.

## 2. Results

### 2.1. Effect of Anthracene on M. polymorpha Biomass and Anthracene Accumulation on the Tissues

**Figure 1:**
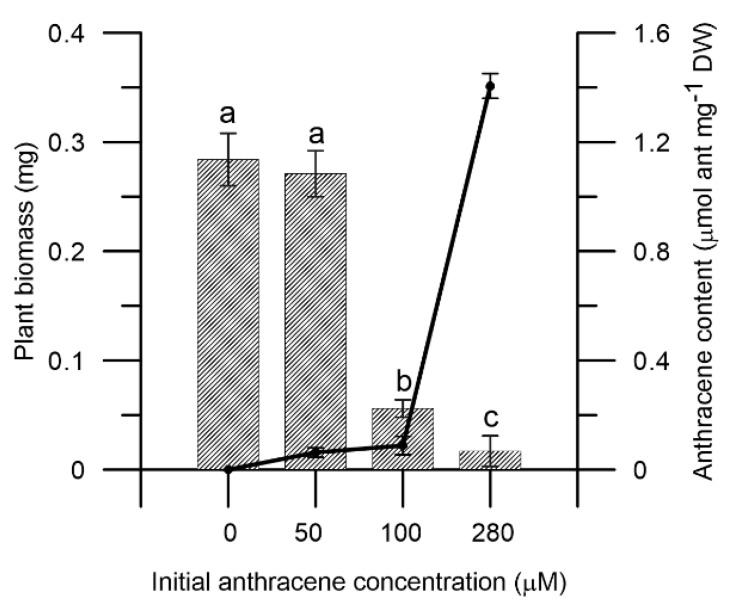
Biomass (bar plot), anthracene content (line plot) in plant tissues of *M. polymorpha* in vitro experiments in minimum medium supplemented with 50, 100 and 280 µM of anthracene. The data represent the mean ± SE (standard error, *n* = 3). Values with the same letter are not significantly different between treatments (*p* ≤ 0.05), as determined by Tukey’s test.

*M. polymorpha* plants showed a clear sensitivity to increasing concentrations of anthracene (Figure 1). When the plants were exposed to the pollutant, a decrease in the final biomass was observed in the presence of 100 and 280 µM (0.056 mg ± 0.008 and 0.017 mg ± 0.014, respectively) of anthracene with respect to the control (0.284 mg ± 0.024), resulting in a significant decrease of plant dry weight (80% and 94%, respectively) (Figure 1). The accumulation of anthracene in plant thallus was also analysed. The absorption on plant tissue increased when increasing anthracene concentration. No significant differences were observed at 50 and 100 µM of anthracene, and 1.4 µmol anthracene mg^−1^ plant dry weight was detected at 280 µM of anthracene (Figure 1). Our results support a high coefficient of regression confirming that anthracene absorption within the thallus depends on the media concentration of the pollutant (R^2^ = 0.191, (*p* ≤ 0.001)).

### 2.2. Effect of Anthracene on the Photosynthetic Apparatus and Chloroplast Ultrastructure

**Figure 2:**
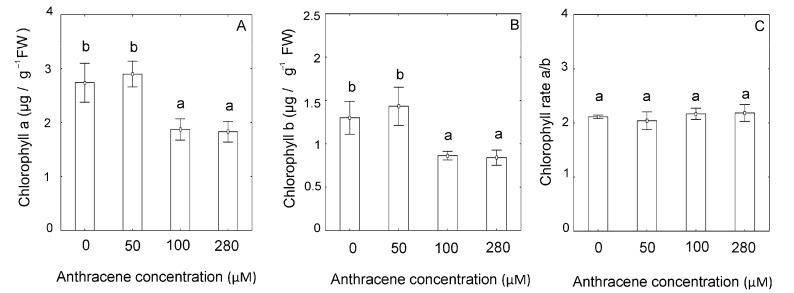
Chlorophyll a content (**A**); chlorophyll b content (**B**); Chlorophyll a/b ratio (**C**) of *M. polymorpha* in the presence of the anthracene (0, 50, 100, 280 µM). The data represent the mean ± SE (standard error, *n* = 3). Values with the same letter are not significantly different between treatments (*p* ≤ 0.05), as determined by Tukey’s test.

**Figure 3:**
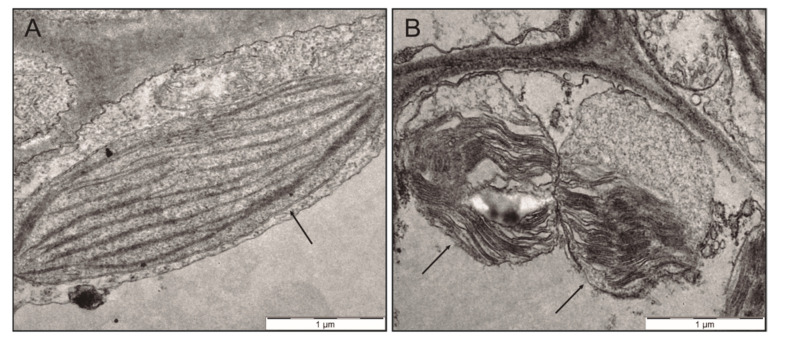
Transmission electron microscopy (TEM) of *M. polymorpha* chloroplast growing in the absence (**A**) and in the presence (**B**) of 280 µM of anthracene. Arrows indicate chloroplasts in the control and chloroplast membrane alteration in 280 µM treatment.

The content of chlorophyll *a* and *b* followed a monophasic behaviour typical of a dose-dependent response of biological systems. Whereas 50 µM slightly induced, but not always significantly, both pigments’ concentrations in plant tissues, higher concentrations of the pollutant (100 µM and 280 µM) strongly repressed their concentration in plant thallus (Figure 2A–C). Although changes in chlorophyll concentrations were observed, no statistically significant changes were measured for Chl *a/b* ratio. Only at 280 µM anthracene, we observed 23.6% of the chloroplast showed changes in their ultrastructure compared to the control treatment with membrane damage and starch granule’s presence. The abnormal organization of the chloroplast anatomy was was associated to the development of large starch granules with thylakoid-granum morphology lacking the smooth elongated shape found in control plants (Figure 3). These ultrastructure changes in the chloroplast were not observed at 50 and 100 µM concentrations.

The net photosynthetic rate did not significantly differ between treatments (Supplementary Table S1). Although the differences detected in the photosynthesis rates were not significant, a strong reduction in the photosynthesis values of the plants treated with anthracene was detected, reducing at least 50%. In addition, the *Fv/Fm* ratio remained constant between treatments (Supplementary Table S1).

### 2.3. Anthracene Treatment Induced ROS-Scavenging Genes

RNA-sequencing (RNA-seq) analyses provided a genome-wide view of anthracene toxicity on *M. polymorpha* thallus. We identified 349 differentially expressed genes: 92 downregulated (*p* value < 0.05, log2(FC) < −1) and 257 (*p* value < 0.05, log2(FC) > 1) upregulated (Supplementary Table S2) upon exposure to anthracene. We identified several protein families within each group of up and downregulated genes (Supplementary Figure S1c). The group of upregulated genes included several late-embryogenesis abundant proteins (LEA) and dehydrin (DHN) genes. To evaluate the overlap of the molecular response induced by anthracene and other abiotic stresses we compared the fold change of differentially expressed genes (DEG) of *M. polymorpha* plants treated with anthracene, ABA [26] and NaCl [27]. We observed a high correlation of the anthracene treatment with NaCl-induced genes (*p* value = 1.3 × 10^−24^) and ABA-induced genes (*p* value = 2.9 × 10^−84^) (Supplementary Figure S1a).

Among anthracene-induced genes, we found an enrichment of polyphenol oxidase (PPO) and dirigent (DIR) protein families involved in secondary metabolism. We detected only a few transcription factors among DEGs, including MpERF20 and the MYC-type MpBHLH4, also induced by the defence related hormone 12-oxo-phytodienoic acid (OPDA) [28,29].

As ROS-scavenging enzymes play a central role in the physiological response to oxidative stress, we studied them in the RNA-seq experiment. First, we annotated all DHAR, MDHAR, APX, CAT, SOD, NOX, GR, PRX and GST enzyme genes in the *M. polymorpha* genome (Supplementary Table S3) and predicted their respective subcellular localization using LOCALIZER [30]. From 246 genes involved in ROS scavenging, only 10 were DEGs (Supplementary Figure S1b). Within this selected group, the anthracene treatment induced MpDHAR1, MpPOD161/159 and MpGST27 and repressed MpCAT4 and several MpPOD including MpPOD26/112/114/115/117/122 (see Supplementary Figure S1d).

We questioned how similar the transcriptional responses to PAHs of *M. polymorpha* and the angiosperm Arabidopsis thaliana were. Therefore, we analysed the group of ortholog genes with a similar response to PAHs comparing the transcriptomic response of anthracene-treated *M. polymorpha* to phenanthrene-treated *A. thaliana* plants [31]. We found a modest overlap (10 genes) of both transcriptomes *(p* value = 0.00015), including orthologs of GST, DHAR and LEA genes that were positively regulated in both PAH treatments (Supplementary Figure S1B).

We also compared the anthracene transcriptional response with DEGs reported in *M. polymorpha* upon UV-B treatment [32] or exogenous application of OPDA [29]. We found a modest overlap with upregulated genes in UV-B (*p* value = 0.0064) and a significant enrichment with OPDA (*p* value = 2.1 × 10^−55^), suggesting a mild cross-talk of anthracene response with other stresses (Supplementary Figure S2). Altogether, the results show a significant impact of anthracene in the transcriptomic landscape related to ROS-detoxifying genes of *M. polymorpha*.

### 2.4. Anthracene Induces Oxidative Damage: Histochemical Staining and Fluorescence Test Determinations

**Figure 4:**
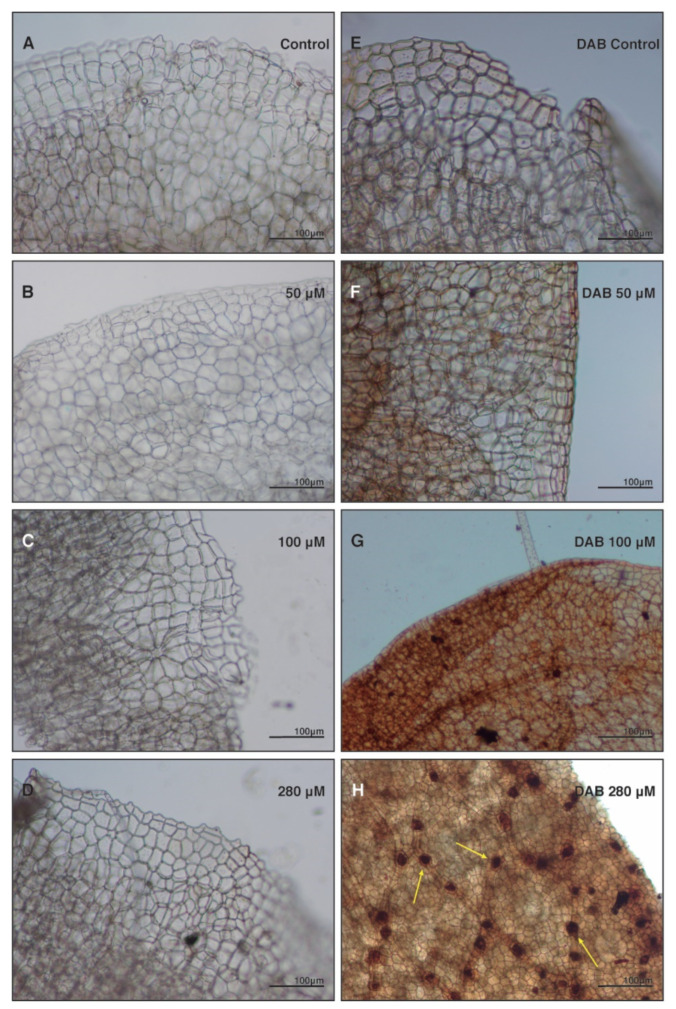
Histochemical analysis with 3,3′-Diaminobenzidine (DAB) as ROS indicator. Thallus of *M. polymorpha* grown in the absence and presence of anthracene. (**A**–**D**) are treatments without DAB staining, and (**E**–**H**) are the treatments with the DAB staining. Arrows indicate assays positive in the polymerisation of DAB in the presence of H_2_O_2_.

**Figure 5:**
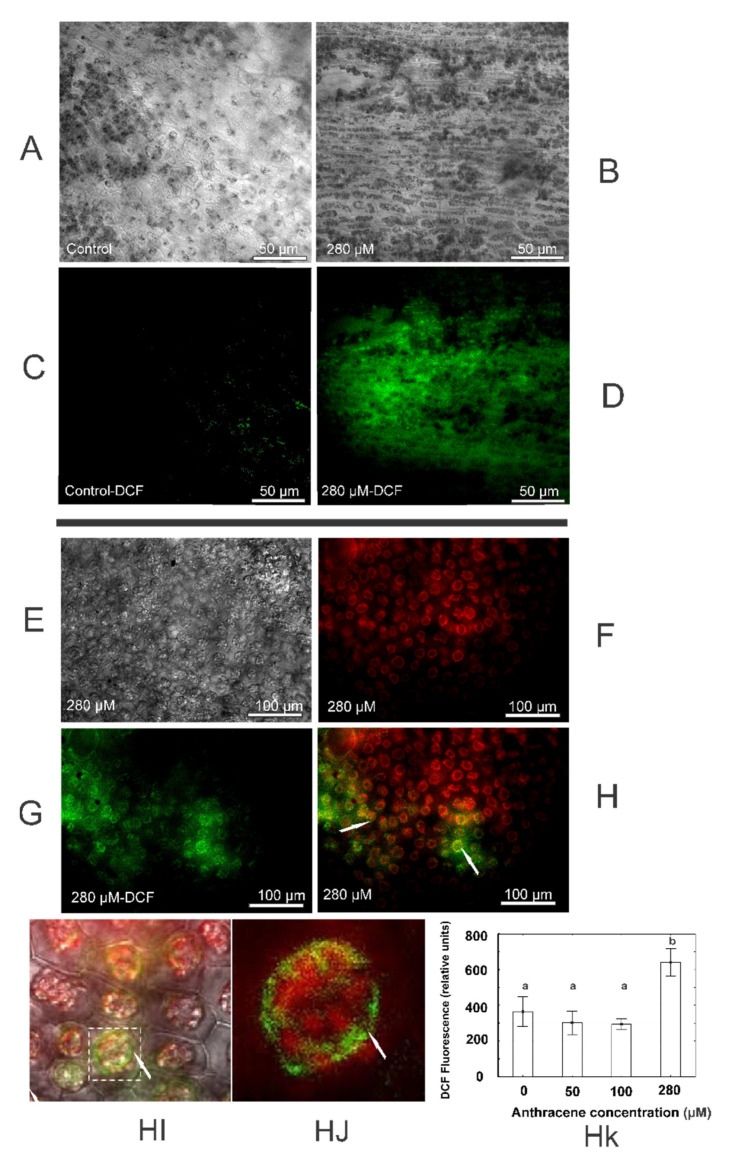
ROS presence in thallus of *M. polymorpha*. Thallus of *M. polymorpha* grown in the absence (**A**) and presence (**B**) of 280 µM of anthracene; (**C**,**D**) are the same treatments after DCFDA staining. Green signals indicate DCF fluorescence. Thallus of *M. polymorpha* in presence of 280 µM of anthracene (**E**), chlorophyll autofluorescence (**F**), DCFDA fluorescence (**G**) overlap of autofluorescence and DCFDA fluorescence micrographies (**H**), green florescence around chloroplast under zoom in H (**HI**,**HJ**) and quantitative oxidation measured as relative units of fluorescence (**Hk**). Red signals indicate chlorophyll fluorescence; green signals indicate DCF fluorescence. The data represent the mean ± SE (standard error, *n* = 4). Values with the same letter are not significantly different between treatments (*p* ≤ 0.05), as determined by Tukey’s test.

The qualitative analysis to test oxidative damage by H_2_O_2_ in the plant using histochemical DAB staining showed strong ROS-related precipitates in anthracene-treated plants (280 µM) (Figure 4). Concomitantly, plants exposed to anthracene showed increased DCF oxidation by the action of intracellular ROS (Figure 5A–D) and a strong green fluorophore emission was observed in comparison to the control in the 280 µM treatments (Figure 5C,D, respectively). The emitted intensity was greater in plants exposed to anthracene compared to the control; 364.5 ± 154.2 and 641 ± 140 DCF fluorescence (relative units), respectively (*p* = 0.009) (see Bar graph Figure 5Hk). In addition, the arrows in the overlap of autofluorescence and DCFDA fluorescence micrographies (Figure 5H,HI) show that ROS are specifically located in the chloroplast aggrupation and around them.

### 2.5. Analysis of Oxidative Damage at the Enzymatic and Lipid Peroxidation Levels

**Figure 6:**
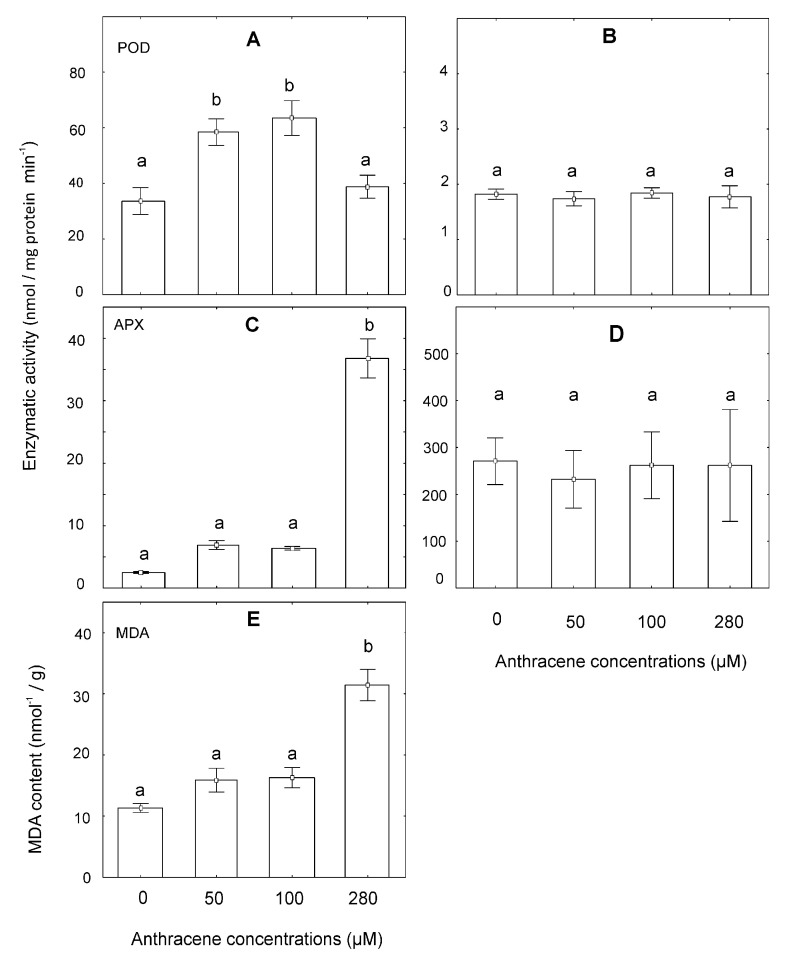
Effect of various anthracene concentrations on the activity of (**A**) Peroxidase (POD); (**B**) Superoxide dismutase (SOD); (**C**) ascorbate peroxidase (APX); (**D**) Catalase and (**E**) malondialdehyde (MDA) content. The data represent the mean ± SE (standard error, *n* = 6). Values with the same letter are not significantly different between treatments (*p* ≤ 0.05), as determined by Tukey’s test.

SOD and CAT enzymes did not show significant differences with respect to the control treatments. On the other hand, POD and APX enzymatic activity increased compared to control plants. POD activity was significantly higher in the presence of 50 µM and 100 µM of anthracene than in control plants (Figure 6A). In addition, a greater activity of APX was registered in plants grown in 280 µM of anthracene (Figure 6B). Related to MDA content, plants grown in 280 µM of anthracene reached values almost three times greater (Figure 6E).

## 3. Discussion

In this study, we documented the physiological effects at the biochemical and molecular level of high doses of anthracene on *M. polymorpha* L. plants. The ability of this plant to tolerate adverse environmental conditions and to colonize contaminated soils and wetlands could be related to the physiological response described in this study.

As indicated above, concentrations of more than 500 µM of anthracene have been recorded in nature. In this study, *M. polymorpha* L. grew within the entire range of tested concentrations, although a loss of biomass was recorded. Previous studies with a different PAH using smaller concentrations in the order of 10 µM, showed that the liverwort *Riccia fluitans* L., when exposed to phenanthrene, was also affected [33]. Thus, this indicates, that *M. polymorpha* has a high resistance to this type of contaminant when compared to R. *fluitans,* other liverwort plants, and this characteristic could be related to the ability of this plant to colonize contaminated or altered places [33]. Although the experimental conditions of our tests are not the same as other studies carried out with plants and PAHs, *M. polymorpha* tolerated higher anthracene concentrations than some vascular plants. For example, with respect to anthracene, [34] tested concentrations of anthracene up to 0.04 µM in leaves of lettuce and radish plants, while [35] tested concentrations of up to 240 µM in carrot roots. The authors of [13] tested concentrations between 40 µM to 1 mM in Arabidopsis using a different PAH such as phenanthrene. This indicates that the tolerance is intrinsic to the plant, the compound used and the study system.

The presence of anthracene in the medium strongly inhibits the growth of *M. polymorpha* and decreases the plant biomass (Figure 1), which is a typical stress response-like symptom observed in plants. Thereby, the detection of anthracene in the tissues correlates with the amount of pollutant in the medium, showing that anthracene accumulates in the cells. This fact supports the hypothesis of previous studies that until now have not been tested [19], confirming that *M. polymorpha* is able to accumulate, maybe passively, anthracene in their tissues. Although we cannot establish whether the internalization of the contaminant is passive or active, our microscopic assays indicate that anthracene or part of it accumulates in the cell walls of *M. polymorpha* and in the chloroplast. In bryophytes, the transport route of nutrients and contaminants is apoplastic and follow the same route as the circulation of water [36]. In vascular plants, simple diffusion and aquaglyceroporins may be involved in the passive uptake of PAH, and active uptake is mediated by a phenanthrene/H^+^ symporter [37]. Although these processes are unknown in liverworts, the internalization mechanisms of contaminants could be similar to those described for vascular plants [19]. Interestingly, some transporter-encoding genes are upregulated in our transcriptomic analysis, although further studies are needed to confirm their involvement in anthracene uptake.

A previous report hypothesized that the presence of anthracene could induce variations in the total content of photosynthetic pigments causing a decrease in plant biomass [19]. In the present study, it was also observed that, although the amount of chlorophylls (*a* and *b*) decreased, the rate between both types of chlorophylls remained constant. These results are in agreement with previous studies in vascular plants that show an alteration in the concentration of chlorophylls in the presence of PAHs [13,38]. PSI and PSII exclusively contain chlorophyll *a*, while the light harvesting complex (LHC) has both chlorophylls *a* and *b*, plus some accessory pigments. Therefore, any variation in the a to b chlorophyll ratio indicates a decrease in the efficiency of light collection [39]. Interestingly, our results suggest that the chlorophylls ratio found is at least adequate or sufficient for a functional photosynthesis in our experimental conditions, which was accompanied by values of net photosynthetic rates and *Fv/Fm* comparable to those found in control plants. These results are opposed to those found in vascular plants, where those variations produced a decrease in the rate of photosynthesis [38,40]. The differences in results between the studies could be due to two principal reasons. On the one hand, the measurements were conducted at single time points and longer periods of exposure to the contaminant could generate effects on the photosynthetic apparatus or, on the contrary, acclimatization and recovery of the *Fv/Fm* was not tested in our experiments. It has even been observed that *Riccia* sp. plants exposed to 0.5 µM of phenanthrene showed almost a full recovery of *Fv*/*Fm* in time [33]. Therefore, the proposal of experiments taking several points in time may be the subject of future studies. Interestingly, in our experiments, chlorophylls showed a deficit of around 30 percent, similar to the number of affected chloroplasts, although they showed altered ultrastructure, and were photosynthetically active. Although the differences detected in the photosynthesis rates were not significant, it is important to note that a strong tendency to reduce the photosynthesis values of the plants treated with the anthracene dose was detected, reducing it to at least 50%. A reduction of two units in the photosynthesis rate should be taken as something significantly different from a biological point of view.

Studies in vascular plants suggested that the accumulation of PAHs in thylakoid membranes induces conformational changes and alters some physiological and biochemical processes, membrane permeability and photosynthesis [41,42,43]. As previously observed in vascular plants, the presence of anthracene alters the ultrastructure of *M. polymorpha* chloroplasts, as observed by TEM (Figure 3). These results clearly show that the interference of these processes are conspicuous, specifically in vascular plants. Our experiments suggest that changes in the ultrastructure of the chloroplasts do not modify the photosynthesis, indicating that the balance of the photosynthetic pigments is not related directly with the change observed in the ultrastructure.

Alterations in the ultrastructure of the chloroplasts are consistent with enhanced lipid peroxidation, due to oxidative damage that is induced by anthracene toxicity shown by MDA, which increased by 3-fold. This damage was not sufficient enough to be detectable on PSII health (*Fv/Fm* ratio), which would affect net photosynthesis. These could be partially explained by several factors. As explained above, not all chloroplasts were affected by anthracene toxicity, those that were affected did not lose their metabolic function of photosynthesis, although they did show alterations, including altered thylakoid-granum and large starch granules. Altogether, our data indicate that the decrease in growth observed in the experiments performed by [19] was not caused by an alteration in carbon fixation in the photosynthetic process. A resource allocation towards other physiological processes, such as the activation of ROS-scavenging enzymes or even the effort to balance the loss of the chloroplast portion, would explain, at least in part, the strong growth inhibition induced by anthracene toxicity. However, it is clear that we cannot rule out that there are other processes in which they could be occurring.

The transcriptomic analysis of anthracene-treated plants supports the activation of some ROS-detoxifying genes and, simultaneously, the induction of several abiotic stress related genes such as *LEA* and *DHN* genes, well known maker genes of the abiotic stress response of *M. polymorpha* controlled by the phytohormone abscisic acid (ABA) [26,44,45]. In legumes, LEA proteins play their physiological role through protein protection, membrane stabilization and ion sequestration [46]. Other protective functions are also plausible since in vascular plants LEA enhances photosynthetic efficiency and reduces ROS levels under drought stress [47]. Interestingly, the molecular fingerprint of anthracene in *M. polymorpha* plants resembles the transcriptomic response of *M. polymorpha* plants treated with ABA or NaCl, while the loss-of-function allele of the ABA receptor, the *pyl* mutant, shows the opposite pattern. These results suggest that anthracene, and maybe other abiotic treatments as well, induces a common response shared by different abiotic stimuli that might largely rely on an intact ABA signalling pathway of *M. polymorpha* and help to understand the acclimation response of liverworts to PAH and other stress responses [48]. Moreover, the PAH-induced growth repression might be explained by the activation of the ABA signalling pathway, as described in transgenic plants overexpressing the ABA receptor [26]. Anthracene also induced additional phenotypes associated with abiotic stress, such as the production of starch granules into the chloroplast also observed in *M. polymorpha* plants treated with ABA, water deficit and NaCl treatments [49]. In addition, our transcriptomic results suggest that, despite the fact that stress response shows strong singularities in different plant lineages and gene regulatory networks evolve dynamically, there are still conserved responses among plants [48]. We showed that *M. polymorpha* displayed a partially conserved response to environmental pollutants such as *Arabidopsis thaliana*, including LEA and ROS-scavenging genes, accompanied with lineage-specific genes (e.g., PPO, DIR). Among anthracene induced genes, we found an enrichment of PPO and DIR genes involved in secondary metabolism that could also contribute to the antioxidant activity against anthracene toxicity. These gene families are particularly expanded in the genome of *M. polymorpha* and they may have contributed to its singular phenylpropanoid metabolism [28]. Moreover, PPOs, as well as GSTs, may participate in anthracene metabolization, as has been recently proposed in *Ulva lactuca* (Chlorophyta algae) [50]. In the case of DIRs, which are involved in radical coupling in lignin and lignan polymerization reactions [28], they could participate in cell wall reinforcement and thereby exert a protective role.

ROS-scavenging enzymes are the main response in vascular plants to stress caused by PAHs [24,51,52]. The present study supports this hypothesis and shows that this response extends not only to vascular plants. Studies in vascular plants show that high levels of PAH produced toxic ROS that may exceed the capacity of the antioxidant systems of the Arabidopsis plant [13] and, although the response between vascular and non-vascular plants is common, the intensity of the response is specific of the plant. Consequently, they proposed that excess ROS may have caused the observed reductions in plant growth and biomass, as well as damage to the chloroplast ultrastructure [13], as we also observed in our studies. They also stated that photosynthesis presumably decreases under these conditions to a low production of ROS. Other authors have affirmed that excessive levels of ROS in vascular plants exposed to PAHs lead to damaged cell structures and can cause cell death [11,24,53,54]. In this study, the histochemical staining showed an increase in ROS content specially located in the chloroplast and around them. This would indicate the presence of reactive oxygen species produced by the chloroplast that could be released into the cytoplasm when *M. polymorpha* grew in a medium with anthracene.

In the system, anthracene–*M. polymorpha*, the enzymatic activities of POD and APX increased compared to the controls (Figure 6). Interestingly, the enzymatic activity of SOD, POD and APX in phenanthrene-treated Arabidopsis plants was also enhanced [13]. Other authors proved that in different species of vascular plants of agronomic interest, the activity of SOD, POD, APX and CAT is important in phenanthrene-induced stress mitigation [53]. Moreover, the antioxidant activity of photosynthetic pigment is well known [55] and has been described as inhibiting lipid peroxidation [56,57]. In this system, we observed that an increase in lipid peroxidation (Figure 6) was concomitant with a decrease in chlorophyll content (and POD activity) in the presence of anthracene. These results, together with those of other authors, indicate that antioxidant activity is triggered in the presence of PAHs and could vary due to two aspects, the plant species and the type and concentration of PAH in the environment. A thorough understanding of the biochemical and molecular responses to PAHs in different plant lineages will help us to model the impact of anthropogenic action in natural and urban regions.

## 4. Materials and Methods

### 4.1. Chemicals

Anthracene (95% purity, cat. number 31581) was purchased from Sigma-Aldrich (Madrid, Spain). Acetone, n-hexane, acetonitrile and water (HPLC degree) were purchased from VWR (Barcelona, Spain).

### 4.2. Plant Biomass and Treatments

*Marchantia polymorpha* L. used in this study are currently maintained in vitro culture in University of Comahue. Plants were placed into glass flasks containing minimum medium [58] supplemented with anthracene. Based on the growth curves reported in previous studies of [19], spanning the highest anthracene concentration ranges found and used to study the influences of PAHs on different plants PAH-enriched solid culture medium was prepared to reach the final concentrations of 50 µM, 100 µM, 280 µM.

The contaminant was prepared as a stock solution in acetone (5 mM) which was added to the culture media to reach the final concentrations. To evaporate the acetone and avoid the toxic effect, the media was stirred for 15 min (50 °C). The medium without contaminant (PAH free) was used as control [19,35,59]. A minimum of three replicates were used per treatment.

Similar pieces of fresh material of plant growing actively (1 cm^2^) were transferred individually into flask-shaped glass pots (50 mL) to avoid differences in the biomass inoculated between treatments. The base of the flasks was covered with aluminium foil in order to prevent UV-oxidation of anthracene [19]. The plant flasks were placed into a plant growth chamber with a light/dark cycle of 16/8 h and controlled temperature of 25 °C. Photosynthetic photon flux density (PPFD) at 45–60 μmol photons m^−2^s^−1^ was supplied by cool white fluorescent tubes.

The experiment was maintained for 30 days. At this time, plants were harvested by separating the biomass from the medium. Before any treatment, the plants were repeatedly washed to remove traces of the culture medium or contaminant adhered to the tissue surface. One quarter of the plants were preserved immediately in liquid nitrogen for chlorophyll and anthracene content after harvest and preserved at −80 °C. The second quarter of the tissue material was dried in an oven at 80 °C until constant weight to determine the final plant biomass expressed as dry weight (DW) at 30 days. The third quarter was used for histochemical analyses and the last quarter of the tissue material was used for transcriptomic analyses.

### 4.3. Anthracene Analyses

Anthracene was extracted from the plant tissues using a modified protocol of [60]. Briefly, dry plants were homogenised with a mixture of n-hexane–acetone (2:1 v/v) followed by three sonication cycles (30 min at 60 °C). The samples were mixed with 6 mL of HPLC water and vortexed during 1 min. The upper n-hexane layer was pipetted out and evaporated using a rotary evaporator at 40 °C (Heidolph Rotary Evaporator, Laborota 4000). The final residue was re-suspended in acetonitrile and analysed using a High Performance Liquid Chromatograph (Agilent ^®^, Hewett-Packard 1050, Palo Alto, CA, USA) equipped with a diode array detector (DAD; 190–700 nm). Separation of the compounds were carried out using a Synergi Fusion RP C18 column (4 μm particle size, 4.6 mm internal diameter × 150 mm length; Phenomenex^®^, Madrid, Spain) in isocratic mode (85% B) using B: acetonitrile and A: water (0,01% phosphoric acid, pH 2) as mobile phase. The temperature of the column was set at 23 °C and the flow rate was maintained at 0.9 mL min^−1^. Anthracene was quantified at 251 nm. Results were expressed as mmol PAH mg^−1^ dry weight.

### 4.4. Chlorophyll Content

The chlorophyll content (chlorophylls *a* and *b*) was measured by high performance liquid chromatography (HPLC) based on the UV-visible spectrum and retention time. Plants were harvested and 1 mg from each replicate was immediately frozen in liquid nitrogen. Plant samples were ground in liquid nitrogen, re-suspended in 1 mL of cold acetone and centrifuged at 10,000 rpm and 4 °C for 10 min. The supernatant was transferred to a glass vial and injected into the HPLC. Chromatographic analyses were performed on a Waters Delta 600E HPLC System equipped with a photodiode array detector (Waters 2998) and controlled with Empower 2 software. Analytical separations were performed in an Inertsil ODS-2 column (5 μm particle size, 4.6 mm internal diameter × 250 mm lenght). The mobile phase was acetonitrile–methanol–ethyl acetate (60:30:10) with a flow rate of 0.8 mL min^−1^ and a sample injection volume of 100 µL [61]. The online spectra were acquired in the wavelength range of 220–700 nm with a resolution of 1.2 nm. The eluted substances were monitored at 430 and 660 nm. Chlorophylls *a* and *b* were identified using standards (C5753, Sigma-Aldrich, USA C5878, Sigma-Aldrich, USA). Chlorophyll concentrations were expressed based on the plants fresh weight (FW).

### 4.5. Transmission Electron Microscopy (TEM) Analysis

Chloroplast ultrastructure was analysed on mature thallus at 30 days. The thallus was washed in a phosphate buffer (PBS; 0.1 M, pH 7.0), sliced into 10 pieces of 1 to 5 mm and fixed for 3 h in 3% glutaraldehyde in a PBS buffer. The samples were then placed in cool osmium tetroxide (1.5% in PBS buffer) for 2 h. Serial immersions in ethanol (50, 70, 96 and 100%) were used for dehydration, ending with a wash in absolute acetone. After that, the samples were placed in acetone–resin (50:50) for six hours, followed by 100% resin overnight. The inclusion of the samples was made in Spurr resin and polymerised for 48 h at 60 °C. The resin blocks were sliced with an ultra-microtome into ultrathin sections with a thickness of 60 to 70 nm, and stained with 1% uranyl acetate and 1% lead citrate. The cellular structures were observed using a Philips CM200 TEM at 200 kV, and the images were captured with a digital Gatan camera.

### 4.6. Histochemical Stain and Fluorescence Test

In order to establish the potential oxidative damage to cells and tissues, histochemical analyses with diaminobenzidine (DAB) and dichlorodihydrofluoresce in diacetate (DCFDA-SIGMA D6883) were performed. For DAB staining assays, small thallus sections of 1 mm to 5 mm were made and the samples were placed in DAB (pH 7.0, 0.5 g L^−1^) overnight, then dehydrated in 100% ethanol and observed under a light optical microscope. The qualitative damage was observed by the presence of DCF oxidised under a Nikon Eclipse E800 fluorescence microscope with a Leica DC 350FX camera. The emission of DCF oxidised registered in the range of 530 nm using a Synergy™ HTX Multi-Mode Microplate Reader; fluorescence was measured in terms of the intensity of the fluorescence emitted [62]. DCF fluorescence intensity was recorded using plants discs of 5 mm in diameter. Plants grown without the contaminant, but with and without DCFDA, were used as control treatments. The plant discs of each treatment were placed in 96-well microplates and excited at 485 nm. An overlap showing the fluorescence of both chlorophyll and DCF was made without the red light filter to observe the specific location of the ROS.

### 4.7. Thallus Gas Exchange and Thallus-Modulated Fluorescence of Chlorophyll

The net photosynthetic rate was measured in five randomly-selected plants per treatment. A LI-6400 portable photosynthesis measuring system (LI-COR, Lincoln, Nebraska, USA) with a 6400-02B LED source was used, providing a photosynthetic photon flux density (PPFD) of 100 μmol m^−2^ s^−1^. The atmosphere of the chamber was maintained at 20 °C and 60% relative humidity with a CO_2_ concentration of 400 ppm and a flow rate of 250 mL min^−1^, using minimum times for each plant of 4 min with logs every 30 s. Plants were pre-acclimated to chamber conditions for 30 min. The net photosynthetic rate (Anet, μmol CO_2_ m^−2^ s^−1^) and thallus transpiration rate (E, mol H2O m^−2^ s^−1^) were registered.

Additionally, the modulated fluorescence of chlorophyll (Chl) was measured with a Junior pulse amperometric modulation (PAM) fluorometer (Heinz Walz GmbH, Effeltrich, Germany).

Chl fluorescence parameters for the Junior-PAM were defined according to the suggestion of the Junior-PAM Chlorophyll Fluorometer Operator’s Guide. Minimum fluorescence of the dark-adapted state (F0) was determined applying a weak modulated light (0.4 μmol photon m^−2^ s^−1^); maximum fluorescence of the dark-adapted state (Fm) was induced in dark-adapted thallus by a short pulse (0.8 s) of saturating light (around 8000 μmol photon m^−2^ s^−1^).

Prior to fluorescence measurements, plants were maintained in darkness for a period of 30 min. After applying a pulse of low-intensity light, the minimum fluorescence yield (F0) was registered. Next, a saturating high-intensity light pulse was applied to induce temporary closing of photosynthetic reaction centre II (PS II) and record the maximum fluorescence yield (*Fm*). Maximum quantum yield of PSII (*Fv/Fm*) was estimated and expressed as the quantum efficiency of open PSII (ratio of variable to maximum fluorescence; *Fv/Fm*) [63].

### 4.8. Oxidative Stress Enzymes and Lipid Peroxidation

One mg of fresh plants from each treatment were homogenised in a mortar, either in PBS or absolute ethanol, and then centrifuged for 10 min at 4 °C at 6800× g. The oxidative stress enzyme activities, SOD, POD, CAT and APX were measured from the cool PBS buffer supernatants while the presence of malondialdehyde (MDA) was measured from the cool absolute ethanol extract as an estimation of lipid peroxidation. SOD (EC 1.15.1.1) was measured using the Nitro blue tetrazolium (NBT) method [64], whereas POD (EC 1.11.1.7) enzyme activity was estimated by the guaiacol method [65]. The decomposition of hydrogen peroxide in water and hydrogen mediated by CAT (EC 1.11.1.6) was detected at 240 nm using the UV method described by [66]. The drop in absorbance at 290 nm was measured as a function of the APX activity enzyme (EC 1.11.1.11) as described by [67]. Enzyme activities were expressed in nmol mg–1 of protein min–1. Finally, the MDA content was measured following the traditional methods described by [68] and expressed as nmol of MDA per gram of plants.

Enzyme activities were measured using a spectrophotometer (Shimadzu DU-700, Japan). Soluble proteins were determined by the dye-binding microassay using Bradford reagent (Bio-Rad) [69] and bovine serum albumin BSA as standards.

### 4.9. RNA-Seq Analyses

Plants growing under control or anthracene treatments (280 µM) were collected. Total RNA was isolated using RNeasy Plant Mini Kits according to manufacturer’s instructions (Qiagen, Germany). RNA sequencing was performed by Macrogen Inc. (Seoul, Korea). Briefly, ribosomal RNA was removed using the Ribo-Zero rRNA removal kit (plant). Quality was tested using the Agilent 2100 Bioanalyzer (Agilent). Libraries construction was performed using the Truseq stranded mRNA Library kits and sequencing was processed with Illumina Novaseq 6000 platform (40 million paired-ended 150 b reads per sample). Sequence analysis was either performed using AIR software (v.1.0, Sequentia Biotech) or Galaxy [70]. In brief, raw sequence files were first subjected to quality control analysis using FastQC (v.0.10.1) before trimming and removal of adapters with HISAT2 [36]. Reads were mapped onto the *M. polymorpha* v5.1 (http://marchantia.info/) assembly with HISAT2 [71]. Alignment rates were around 70–77% in all samples. HTSeqCount [72] was then used to obtain raw expression counts for each annotated gene and normalized to Transcripts Per Million (TPM) using an R script. The differential-expression analysis was conducted with DESeq2 [73]. Due to disparities among the samples, we replaced the fold change (FC) provided by DESeq2 for the ratio of normalized counts in order to use a less conservative approach. Complementary gene expression studies were performed using a similar analysis on reported RNA-seq experiments (DRR093994-DRR093408, DRR127460-127465) and TPM were plotted as described in the corresponding figure using ComplexHeatmap package in R [74]. The transcriptomic data of UV-B and OPDA treatments of *M. polymorpha* plants were obtained from the published Supplementary Tables [29,32].

Genes were annotated using information nomenclature previously published or available at MarpolBase [75]. Protein Family (PFAM) analysis enrichment was performed as described before [76]. Analysis of orthologs and GO-term enrichment was performed using Dicot PLAZA 4.5 [77].

### 4.10. Statistical Analysis

One-way analysis of variance (ANOVA) was used for data analysis using statistical status 7.0. The differences found using ANOVA were validated by Fisher’s or Tukey’s honestly significant difference test (HSD). *Fv/Fm* was analysed with gamma error distribution generalized linear model (GLM). Fisher’s Exact Test was used for gene list comparisons in transcriptomic experiments, using either the whole *M. polymorpha* v5.1 transcriptome or gene orthologs in common with Arabidopsis.

## 5. Conclusions

The results show that, although anthracene produces a decrease in the biomass of *M. polymorpha* and an alteration in the ultrastructure of almost 25% of the chloroplasts, overall, the photosynthetic net processes are compensated. The POD and APX, rather than SOD and CAT, were more sensitive in the plant response of *M. polymorpha* to the contaminants, showing that the contaminated plant tended to catalyse the process of peroxide to water and oxygen. The effect observed in *M. polymorpha* plants could be mainly due to the activation of the two principal mechanisms of detoxification: (1) an increased accumulation of anthracene in plant tissues and (2) enhanced resource allocation to the activation of the antioxidant system. Therefore, a reduction in photosynthetic processes would not be necessary to account for the decrease in production of ROS, as hypothesised in previous studies. Finally, we hypothesize that these characteristics could be related to the ability of *M. polymorpha* to colonize contaminated environments.

## Data Availability

Not applicable.

## Supplementary Materials

The following are available online at https://www.mdpi.com/article/10.3390/plants10071478/s1, Figure S1: Anthracene induced a similar transcriptome response to abiotic stress related treatments. (A) Heatmap showing the comparison of different RNA-seq of *M. polymorpha* upon different growing conditions: Anthracene (this study), NaCl [27], ABA, MpPYL overexpressor transgenic plants, and Mppyl [26]. Colour indicates log2 fold change (FC) compared with their respective controls (see scale bar). Only DEGs in anthracene are shown (*p* < 0.01, |FC| > 1). Genes are ordered by FC observed upon anthracene treatments. (B) Orthologous gene comparison using Dicot PLAZA 4.5 [77] of anthracene upregulated genes with genes upregulated after 24 h of phenanthrene treatment in *A. thaliana* [31]. (C) Top PFAM enriched in among anthracene upregulated genes. (D) Expression values for ROS-related genes; Figure S2: (A) Venn diagram representation of the transcriptomic profiles comparison of upregulated genes in *M. polymorpha* after anthracene treatment a single 12 h high fluence UV-B treatment [32] and 2 h OPDA [29]. (B,C) Top GO enriched terms in anthracene upregulated and downregulated genes. *p*-values indicated Fisher’s exact test results; Table S1: Net photosynthetic rate and chlorophyll fluorescence parameter *Fv/Fm* determinated (Mean values ± SE) on *M. polymorpha* in 0, 50, 100 and 280 µM of anthracene. Net photosynthetic rate was compared with one-way ANOVA (*p* < 0.05) while *Fv/Fm* was compared using Gamma error distribution generalized linear model (GLM, Pchisq < 0.05); Table S2: DEGs of anthracene-treated *Marchantia* plants in comparison with previous studies including: NaCl [46], ABA, MpPYL overexpressor transgenic plants, and Mppyl loss-of-function mutant plants [26]; Table S3: List of manually annotated peroxidase genes following the current nomenclature guidelines for *M. polymorpha*. The table includes Locus ID (*Marchantia* genome v5), Gene symbol, classification and predicted subcellular localization.
