# Supplementary material for: ROS-Scavenging Enzymes as an Antioxidant Response to High Concentration of Anthracene in the Liverwort Marchantia polymorpha L
Source: Plants (Basel). 2021 Jul 19;10(7):1478. doi: 10.3390/plants10071478 (PMC8309224; doi:10.3390/plants10071478)
Supplement: Supplementary file 1 [file plants-10-01478-s001.zip › Supplementary table 1.pdf]

Net photosynthetic rate and chlorophyll fluorescence parameter F_v/F_m (Mean values ±SE) determinated on *M. polymorpha* in 0, 50, 100 and 280 µM of anthracene. Net photosynthetic rate was compared with one-way ANOVA (p<0.05) while F_v/F_m was compared using Gamma error distribution generalized linear model (GLM, Pchisq<0.05).

Parameters	Anthracene concentration (µM)				*p*
	0	50	100	280	
Net Photosynthesis (µmol CO_2 m^{-2} s^{-1})	4.115± 0.982	4.245± 0.681	1.728± 0.519	2. 40±0.537	0.061
F_v/F_m	0.637±0.010	0.606±0.015	0.627±0.009	0.631±0.001	0.115
