# Supplementary figures and images for: ROS-Scavenging Enzymes as an Antioxidant Response to High Concentration of Anthracene in the Liverwort Marchantia polymorpha L
Source: Plants (Basel). 2021 Jul 19;10(7):1478. doi: 10.3390/plants10071478 (PMC8309224; doi:10.3390/plants10071478)

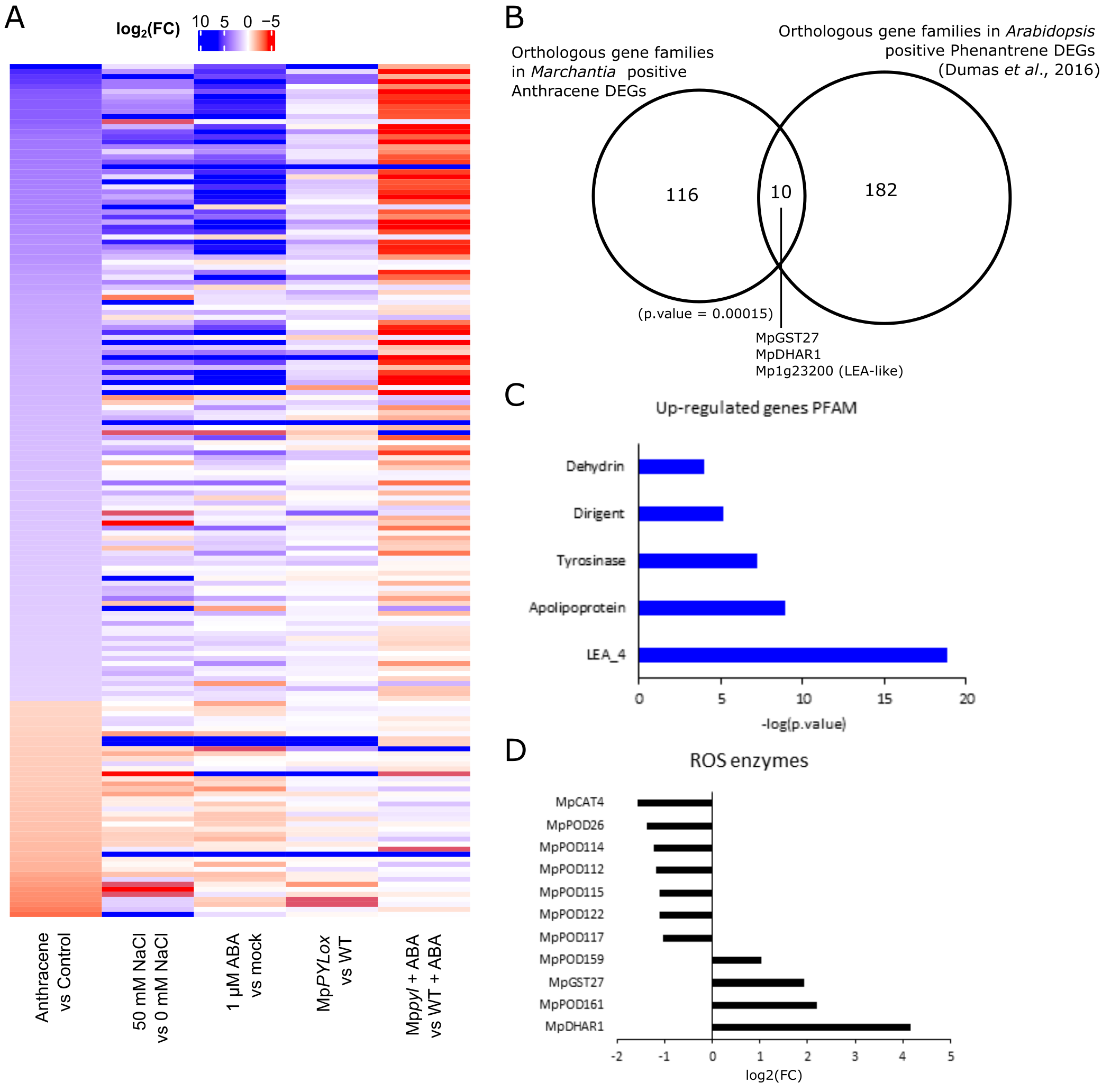

Supplement: Supplementary file 1 [file plants-10-01478-s001.zip › Supplementary figure 1.png]

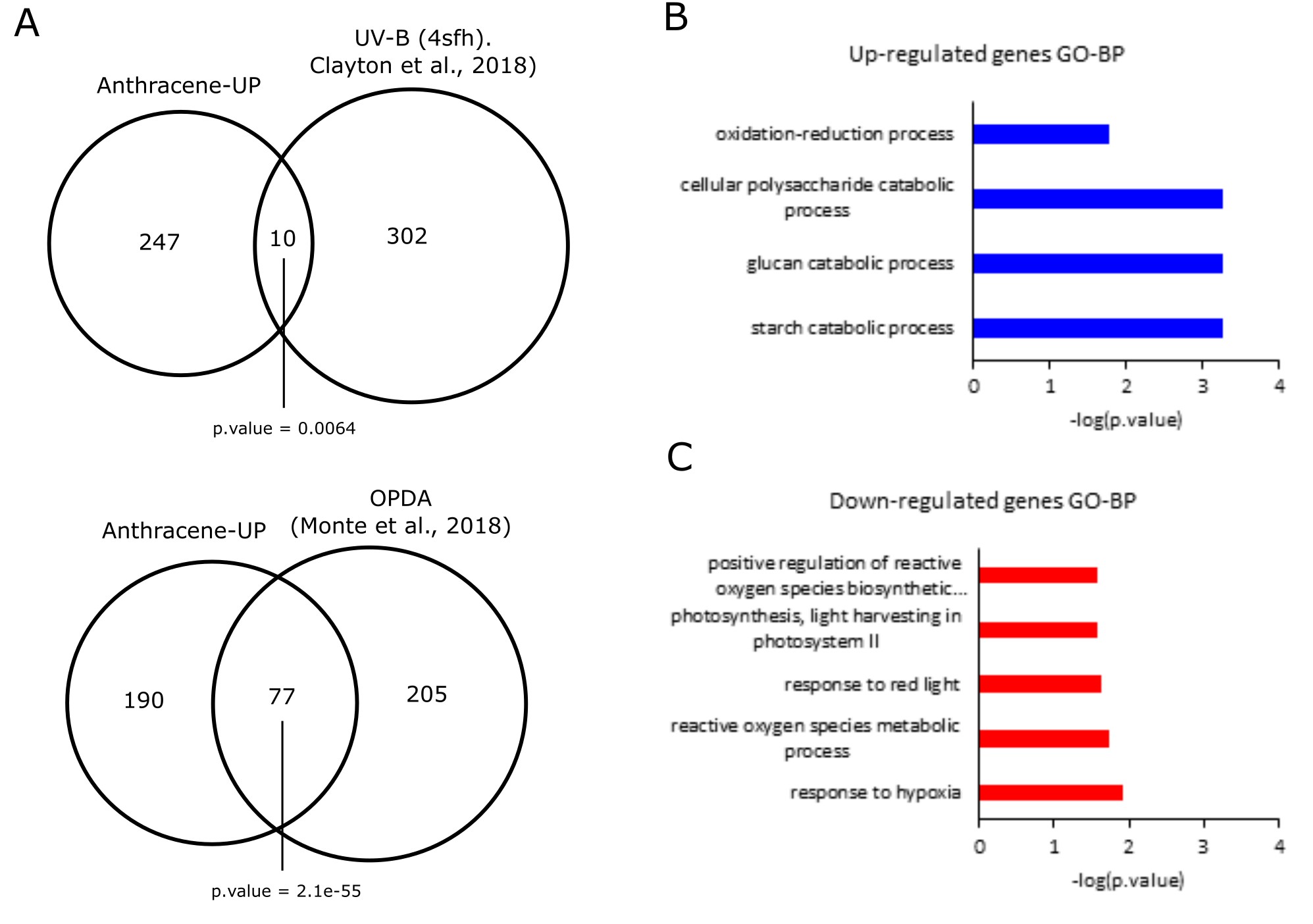

Supplement: Supplementary file 1 [file plants-10-01478-s001.zip › Supplementary figure 2.jpg]
